# The microRNA-6510 as a potential tumor suppressor in head and neck cancer

**DOI:** 10.1038/s41598-025-86796-0

**Published:** 2025-02-18

**Authors:** Agnieszka Sobecka-Giel, Kamila Ostrowska, Wojciech Golusinski, Wiktoria M. Suchorska, Michal M. Masternak, Pawel Golusinski

**Affiliations:** 1 Department of Head and Neck Surgery, Poznan University of Medical Sciences, Poznan, 61-701 Poland; 2 Radiobiology Lab, Department of Medical Physics, Greater Poland Cancer Centre, Poznan, 61-866 Poland; 3 Department of Head and Neck Surgery, Greater Poland Cancer Centre, Poznan, 61-866 Poland; 4 Department of Electroradiology, Poznan University of Medical Sciences, Poznan, 61-866 Poland; 5 Burnett School of Biomedical Sciences, College of Medicine, University of Central Florida, Orlando, FL 32827 USA; 6 Department of Otolaryngology and Maxillofacial Surgery, University of Zielona Gora, Zielona Gora, 65-417 Poland

**Keywords:** Head and neck cancer, Head and neck squamous cell carcinoma, MicroRNA, MiR-6510, Tumor suppressor, Molecular marker, Molecular therapy, Head and neck cancer, miRNAs

## Abstract

Head and Neck Squamous Cell Carcinoma (HNSCC) is the sixth most common cancer worldwide, with approximately 830,000 new cases and 430,000 deaths reported annually. Due to their heterogeneity, these neoplasms differ in their clinical course and response to the therapy. Therefore, it has become imperative to identify specific biological molecules that can potentially establish novel prognostic markers or targets for molecular therapy of HNSCC. MicroRNAs are a class of short, non-coding RNAs that function as post-transcriptional regulators of genes expression. They have been shown to be directly involved in oncogenesis, acting as tumor suppressors or oncogenes. Our previous study demonstrated that miRNA hsa-miR-6510-3p is significantly downregulated in tumor tissue compared to histologically normal tissue from HNSCC patients. Its significant downregulation in tumor tissue is associated with lower chances for recovery and patient’s survival. This study aimed to determine the biological role of miR-6510-3p in HNSCC pathogenesis and its impact on biological processes occurring in cancer cells such as cell cycle, cell proliferation, migration or induction of cell death. We have also examined the impact of the miR-6510-3p on expression of cancer stem cell phenotype markers as well as on sensitivity of HNSCC cells to ionizing radiation. We observed that transfection of HNSCC cells with hsa-miR-6510-3p causes the cell cycle arrest in G2/M phase and is associated with a decrease of cell proliferation, migration and colony-forming ability of cancer cells. We have also demonstrated that hsa-miR-6510-3p induces cell death, increases the sensitivity of HNSCC cells to ionizing radiation and causes a loss of the stemness properties responsible for the occurrence of metastases and relapses of the disease. These results indicated the importance of miR-6510-3p as a marker and a driver of HNSCC disease.

## Introduction

Head and Neck Squamous Cell Carcinoma (HNSCC) is the sixth most common cancer worldwide, with approximately 830,000 new cases and 430,000 deaths reported annually^1^. It primarily affects stratified squamous epithelium of the upper respiratory and digestive tract and due to its anatomical locations, including the oral cavity, nasal cavity with paranasal sinuses, pharynx, and larynx, it represents a clinically heterogeneous group of tumors^2^. The main risk factors for oral and laryngeal cancer include tobacco use and alcohol consumption, while oropharyngeal tumors are frequently associated with human papillomavirus (HPV) infection with particular emphasis on the oncogenic types HPV16 and HPV18^3–5^. Currently, the treatment of HNSCC is based on the anatomical subsite of the tumor, stage of the disease and general medical condition of the patient. The multidisciplinary treatment protocols involve surgical excision, radiotherapy, chemotherapy, and immunotherapy. Despite significant progress in refining these therapeutic strategies in recent decades, the overall 5-year survival rates for patients with head and neck cancer have not significantly improved and average around 50%^3,6^. This is associated with the fact that HNSCC represents a highly heterogeneous disease entity with a diverse etiology and molecular profile^7^. Therefore, it has become imperative to enhance our comprehension of the biology of head and neck tumors, identify novel, specific biological markers, and pinpoint molecules that potentially could serve as targets in molecular therapy for HNSCC. Thus, here we focus on microRNAs (miRNAs) as potential novel markers and future therapeutic targets. miRNAs are a class of short (18–25 bp), non-coding RNAs, which are encoded in the genome as long primary transcripts called primary-miRNAs (pri-miRNAs). These are embedded either as separate transcriptional units or within the introns of protein coding genes. MiRNAs through interactions with the 3’ untranslated region (3’ UTR) of messenger RNA (mRNA) contribute to the regulation of gene expression at the posttranscriptional level. MiRNAs have been demonstrated to modulate the expression of more than 60% of human protein-coding genes^8^. They are involved in various physiological processes such as apoptosis, aging, and the cell cycle, and are implicated in the initiation and progression of numerous pathological conditions^9–11^.

There is also growing evidence that dysregulation of miRNA expression, stemming from genetic mutations, epigenetic alterations, modifications in biogenesis, altered transcription factors expression, or changes in target sites, may contribute to the progression of cancer^12,13^. Comprehensive genome-wide studies profiling of miRNA expression with high-throughput technologies have revealed that nearly all types of cancer, including HNSCC, exhibit a distinctive pattern of upregulated and downregulated miRNAs. Some of these miRNAs, such as miR-30b and miR-150, have been identified as tumor suppressors^14–16^, while others, like miR-21 and miR-205, act as oncogenes^17,18^. It was shown that miRNAs do not play an exclusively cell autonomous role as they have been detected in various body fluids, including tears, saliva, urine, breast milk, and serum. Several studies showed that exosomal secreted miRNAs contribute to intercellular communication, influencing gene expression in both neighboring and distant target cells. Consequently, they can be considered as reliable, non-invasive biomarkers in cancer detection and prediction of therapy response^19–24^.

Our team investigated several different approaches to study miRNAs as potential biomarkers in plasma, tumor and healthy tissue of the HNSCC patients. We have identified numerous miRNAs classified as potential oncomirs, representing miRNAs associated with cancer progression or the opposite ones known as tumor suppressors. Notably, our random forest survival analysis of the The Cancer Genome Atlas HNSC dataset from 497 HNSCC patients narrowed down the extensive pool of miRNAs to 11 upregulated (e.g., miR-196b-5p, miR-1269a) and 9 downregulated (e.g., miR-376c-3p, miR-378c) miRNAs, correlating with clinical outcomes. Furthermore, optimal random forest analysis including 6 different variables such as age at diagnosis, sex, N stage, pathologic stage, gender and race brought our attention to one particular miRNA, miR-6510-3p^25^. This miRNA, which had been understudied in HNSCC and other malignancies, has been found to be significantly downregulated in tumor tissue compared to histologically normal tissue from the same patients. Importantly, patients’ survival analysis revealed that lower expression of miR-6510-3p in tumor tissue is significantly associated with lower chances for recovery and survival^23^.

Following these initial data, in this work we aimed to determine the biological role of miR-6510-3p in head and neck squamous cell carcinoma pathogenesis. To achieve this objective, we developed an experimental HNSCC model by transfection of hsa-miR-6510-3p mimic, its inhibitor and control (scramble) miRNA into two established head and neck cancer cell lines including FaDu and H103. We assessed the transfection efficiency, and we performed follow-up analyses of miR-6510-3p impact on biological processes occurring in cancer cells such as cell cycle, cell proliferation, migration or induction of cell death. We have also examined the impact of the miR-6510-3p on expression of cancer stem cell phenotype markers as well as on sensitivity of HNSCC cells to ionizing radiation.

## Results

### Efficiency of hsa-miR-65103-3p transfection of HNSCC cancer cell lines, Fadu and H103

**Fig. 1:**
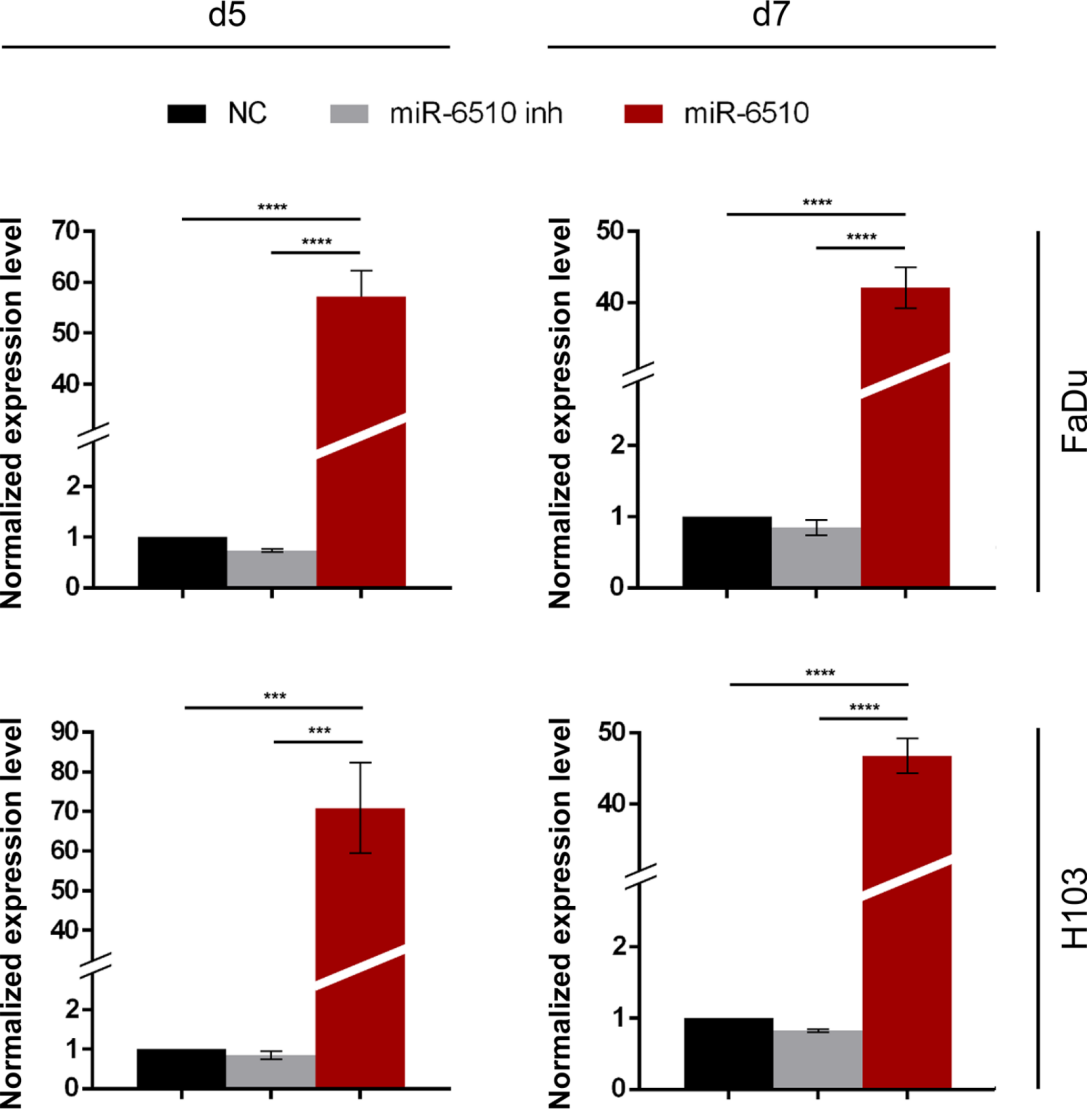
Hsa-miR-6510-3p expression in FaDu and H103 cell lines. NC – cells transfected with non-specific, control miRNA; miR-6510 inh – cells transfected with hsa-miR-6510-3p inhibitor; miR-6510 - cells transfected with hsa-miR-6510-3p; d5 – cells analyzed on the 5th day after transfection; d7 – cells analyzed on the 7th day after transfection. Data are presented as the mean ± standard deviation. ***p < 0.001, ****p < 0.0001 with comparisons indicated by lines.

Selected in this study FaDu and H103, the two major head and neck squamous cell carcinoma cell lines, were subjected to RT-PCR analysis at 5th and 7th day after transfection with hsa-miR-65103-3p mimic, inhibitor and negative control. The analysis indicated significant changes in both FaDu and H103 cell lines. In FaDu cells miR-6510-3p level was 57-fold increased on the 5th day after transfection (*p* < 0.0001) and 42-fold upregulated 7 days after transfection (*p* < 0.0001) compared to control cells (Fig. 1). In H103 cell line transfection with miR-6510-3p mimic resulted in 71-fold increase of miR-6510-level 5 days after transfection (*p* < 0.0001) and 47-fold upregulation on the 7th day (*p* < 0.0001) when compared to control cells (Fig. 1).

### Impact of hsa-miR-6510-3p on cell cycle

**Fig. 2:**
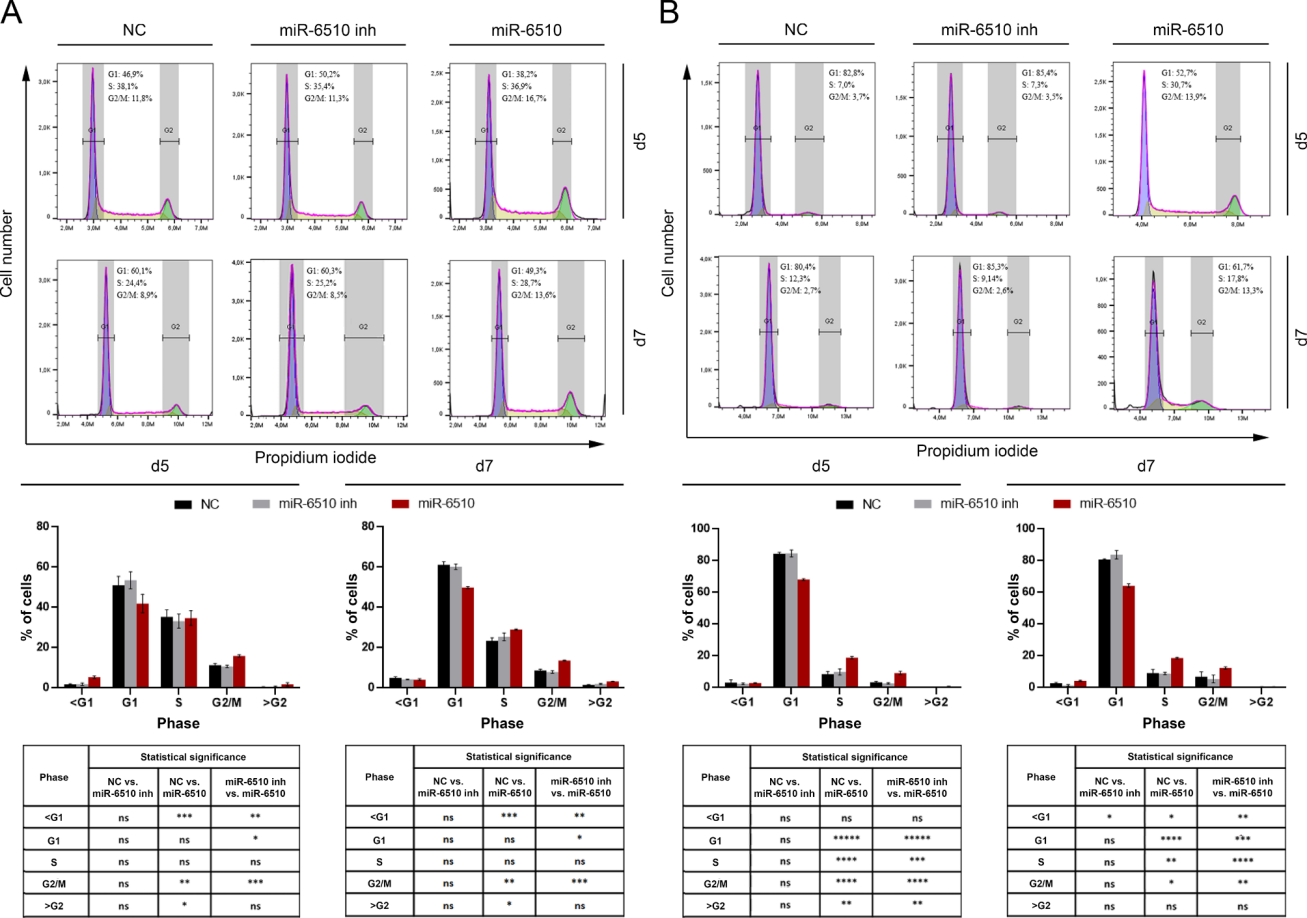
Impact of hsa-miR-6510-3p on cell cycle of (**A**) FaDu and (**B**) H103 cells. Flow cytometry and statistical analysis. Data presented as percentages of cells in the G1, S, and G2-M phases. NC – cells transfected with non-specific, control miRNA; miR-6510 inh – cells transfected with hsa-miR-6510-3p inhibitor; miR-6510 - cells transfected with hsa-miR-6510-3p; d5 – cells analyzed on the 5th day after transfection; d7 – cells analyzed on the 7th day after transfection. Data are presented as the mean ± standard deviation. ns – statistically non-significant, *p < 0.05, **p < 0.01, ***p < 0.001, ****p < 0.0001, ****p < 0.00001 with comparisons indicated in the table.

Flow cytometry analysis using propidium iodide in FaDu cell line after five days post transfection with hsa-miR-65103-3p indicated statistically significant increase of the apoptotic cells fraction compared to control cells (from 1.84 to 5.39%; *p* = 0.000899). At the same time point, an increase in the fraction of cells in the G2/M phase was also observed (11.39% vs. 16.00%; *p* = 0.001235). There was also a trend towards decrease in the percentage of the G1 fraction (51.01% vs. 41.99%; *p* = 0.069032) suggesting cell cycle arrest in the G2/M phase. A similar effect was also observed on day 7 after transfection, where the fraction of the cells in the G1 phase decreased from 61.23 to 49.90% (*p* = 0.000288) while the percentage of the cells in the G2/M phase increased from 8.74 to 13.66% (*p* = 0.000115) after transfection with hsa-miR-65103-3p (Fig. 2A).

In H103 cells, an increase of the apoptotic cells fraction compared to the control cells was observed on day 7 after transfection with hsa-miR-6510-3p (2.79% vs. 4.27%; *p* = 0.015589). Cell cycle arrest in the G2/M phase was also observed in H103 cells at both analyzed time points. On day 5 after transfection, in cells transfected with miR-6510-3p, the percentage of cells in the G1 phase decreased from 84.46 to 68.11% (*p* < 0.000001) compared to control cells, with a simultaneous increase in the percentage of cells in the G2/M phase from 3.48 to 9.23% (*p* = 0.000053). On the 7th day after transfection, a decrease of the G1 fraction (80.76% vs. 64.23%; *p* = 0.000016) and a statistically significant increase in the percentage of cells in the G2/M phase (from 6.98% to 12,46%; *p* = 0.037619) was observed compared to the control group (Fig. 2B).

### MiR-6510-3p decreases proliferation of cancer cells

**Fig. 3:**
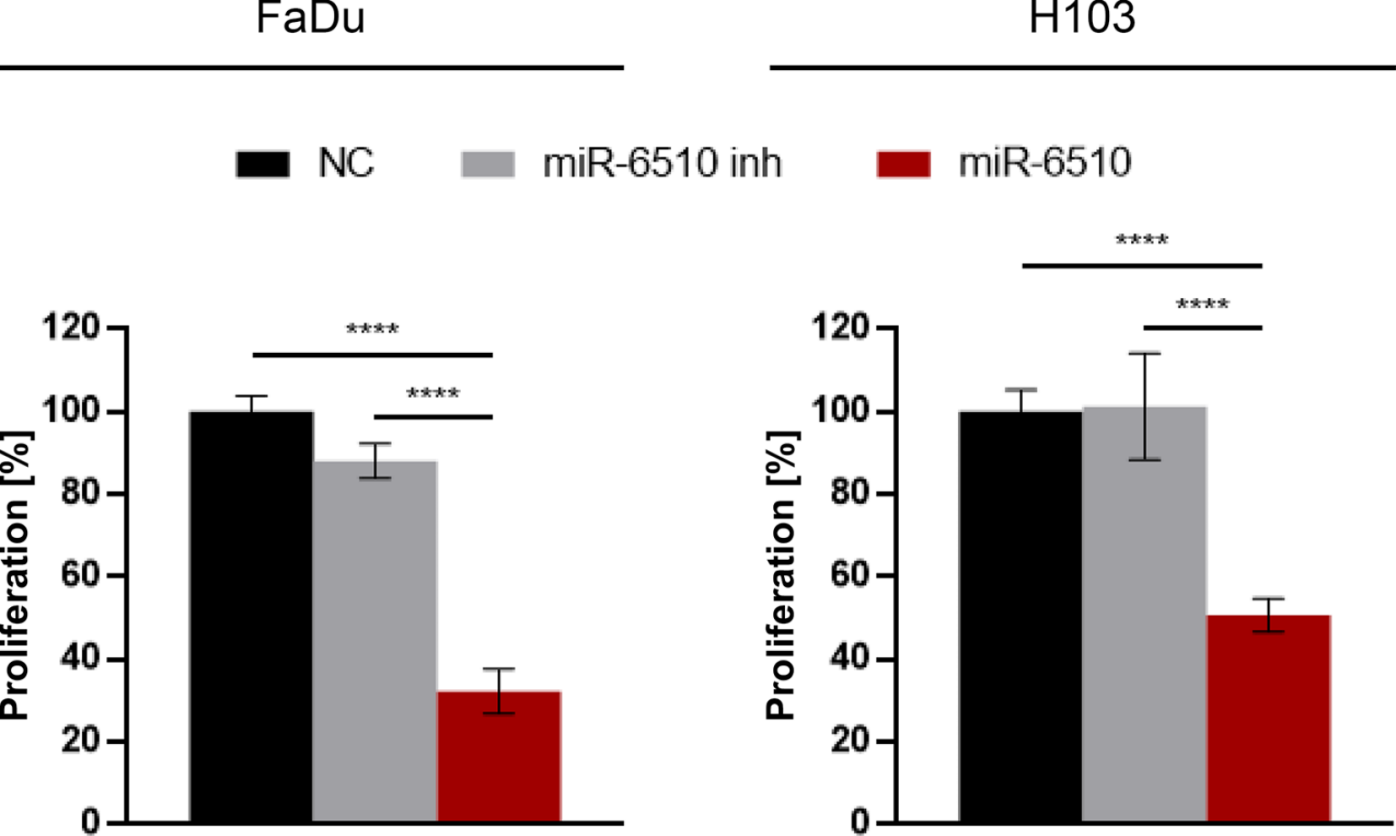
Impact of hsa-miR-6510-3p on proliferation of FaDu and H103 cells. NC – cells transfected with non-specific, control miRNA; miR-6510 inh – cells transfected with hsa-miR-6510-3p inhibitor; miR-6510 - cells transfected with hsa-miR-6510-3p. Data are presented as the mean ± standard deviation. ****p < 0.0001 with comparisons indicated by lines.

The MTT (3-[4,5-dimethylthiazol-2-yl]-2,5-diphenyltetrazolium bromide) cell proliferation assay in FaDu cells indicated that transfection with miR-6510-3p caused significant suppression of cellular proliferation when compared to control and inhibitor transfected cells (32.33%; p = < 0.0001 and 88.01%; *p* = 0.0219, respctively) (Fig. 3). Also the analysis of H103 cells showed a statistically significant decrease in proliferation in miR-6510 mimic treated cells when compared to the control cells (50.76%; *p* = 0.0002) (Fig. 3).

### MiR-6510-3p affects cancer cell migration

**Fig. 4:**
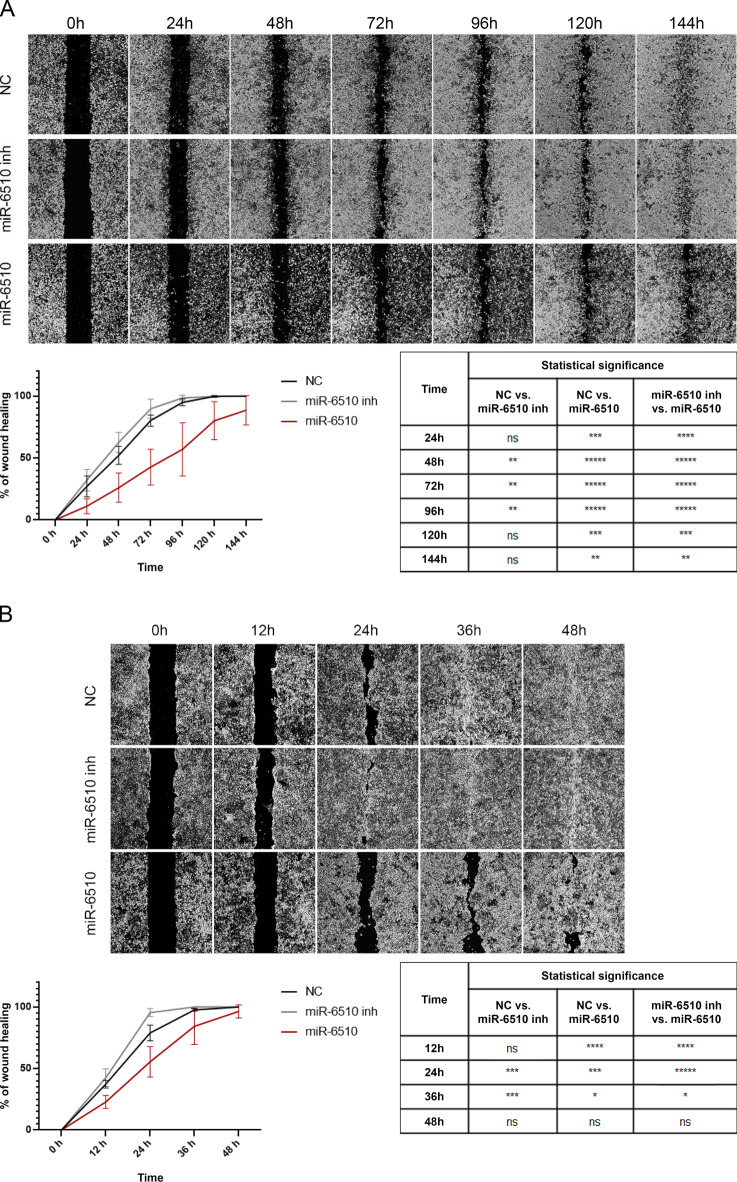
Impact of hsa-miR-6510-3p on (**A**) FaDu and (**B**) H103 cell lines migration ability. Wound healing assay. Zeiss Axio Vert.A1, 20x magnification. NC – cells transfected with non-specific, control miRNA; miR-6510 inh – cells transfected with hsa-miR-6510-3p inhibitor; miR-6510 - cells transfected with hsa-miR-6510-3p. Data are presented as the mean ± standard deviation. *p < 0.05, **p < 0.01, ***p < 0.001, ****p < 0.0001, ****p < 0.00001 with comparisons indicated in the table.

Cell migration using wound healing analysis of FaDu cells revealed a statistically significant reduction in the migration ability at all time points of the experiment in cells transfected with miR-6510-3p compared to control cells (24 h, *p* = 0.000208; 48 h, *p* = 0.000009; 72 h, *p* < 0.000001; 96 h, *p* = 0.000009; 120 h, *p* = 0.000284; 144 h, *p* = 0.003446) and cells treated with miR-6510-3p inhibitor (24 h, *p* = 0.000011; 48 h, p = < 0.000001; 72 h, p = < 0.000001; 96 h, *p* = 0.000003; 120 h, *p* = 0.000289; 144 h, *p* = 0.003446). It was also observed that the motility of cells transfected with miR-6510-3p inhibitor was significantly increased at 48 h (*p* = 0.00294639), 72 h (*p* = 0.0012359) and 96 h (*p* = 0.00212507) time points compared to control group (Fig. 4A).

In H103 cell line the migration ability of the miR-6510 transfected cells was significantly reduced compared to control variant at 12 h, 24 h and 36 h time points (*p* = 0.000042; *p* = 0.000953; *p* = 0.047515, respectively) and also when comparing with cells transfected with miR-6510-3p inhibitor (*p* = 0.000016; *p* < 0.000001; *p* = 0.014313). The motility of cells treated with miR-6510-3p inhibitor was also increased at 24 h (*p* < 0.0001) and 36 h (*p* < 0.0001) time points when compared to control cells (Fig. 4B).

### MiR-6510-3p depletes expression of CD133 cancer stem cell phenotype marker

In order to investigate the effect of hsa-miR-6510-3p micro RNA on the stem cell phenotype of cancer cells, cytometric analysis of the expression of CD44 and CD133 - surface antigens considered as markers of the stemness phenotype in head and neck squamous cell carcinoma was performed.

**Fig. 5:**
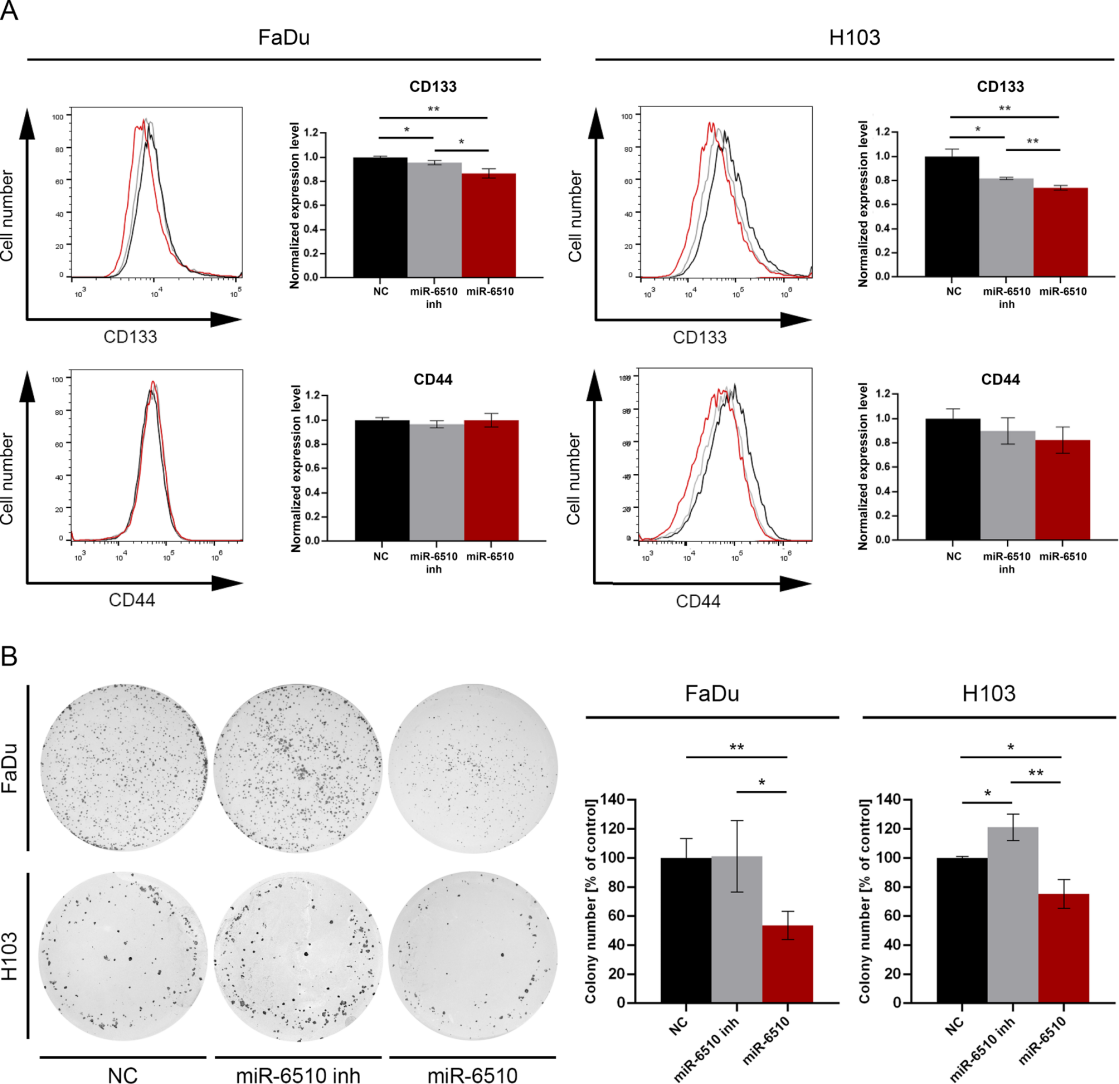
Impact of hsa-miR-6510-3p on the stem cell phenotype markers expression and clonogenic potential. (**A**) Flow cytometry analysis of CD133 and CD44 expression in FaDu and H103 cells. (**B**) Analysis of FaDu and H103 clonogenic cell survival. NC – cells transfected with non-specific, control miRNA; miR-6510 inh – cells transfected with hsa-miR-6510-3p inhibitor; miR-6510 - cells transfected with hsa-miR-6510-3p. Data are presented as the mean ± standard deviation. *p < 0.05, **p < 0.01 with comparisons indicated by lines.

Analysis of CD133 expression showed that transfection of hsa-miR-6510-3p reduces the expression level of this marker in relation to control cells in both the FaDu (*p* = 0.0042; Fig. 5A) and H103 (*p* = 0.0051; Fig. 5A) cell lines. In both cell lines, a decrease in the expression level of this marker was also observed in cells transfected with the miR-6510-3p inhibitor (FaDu: *p* = 0.0189; H103: *p* = 0.0117) (Fig. 5A).

The analysis of the CD44 surface antigen expression did not show statistically significant effect in both cell lines after transfecting with hsa-miR-6510-3p (Fig. 5A).

### MiR-6510-3p reduces colony-forming ability of FaDu and H103 cells

In order to investigate the effect of hsa-miR-6510-3p on the colony-forming ability of HNSCC cells, a clonogenic assay was performed. It was demonstrated that transfection of hsa-miR-6510-3p reduces the clonogenic potential in both the FaDu and H103 cell lines in relation to control cells (*p* = 0.0082 and *p* = 0.0129, respectively) (Fig. 5B) and cells transfected with hsa-miR6510-3p inhibitor (FaDu: *p* = 0.0359; H103: *p* = 0.0042) (Fig. 5B). In H103 cell line an increased colony-forming ability was also observed in cells transfected with the hsa-miR-6510-3p inhibitor compared to the control cells (*p* = 0.0161) (Fig. 5B).

### MiR-6510-3p regulates apoptotic genes in FaDu and H103 cells

**Fig. 6:**
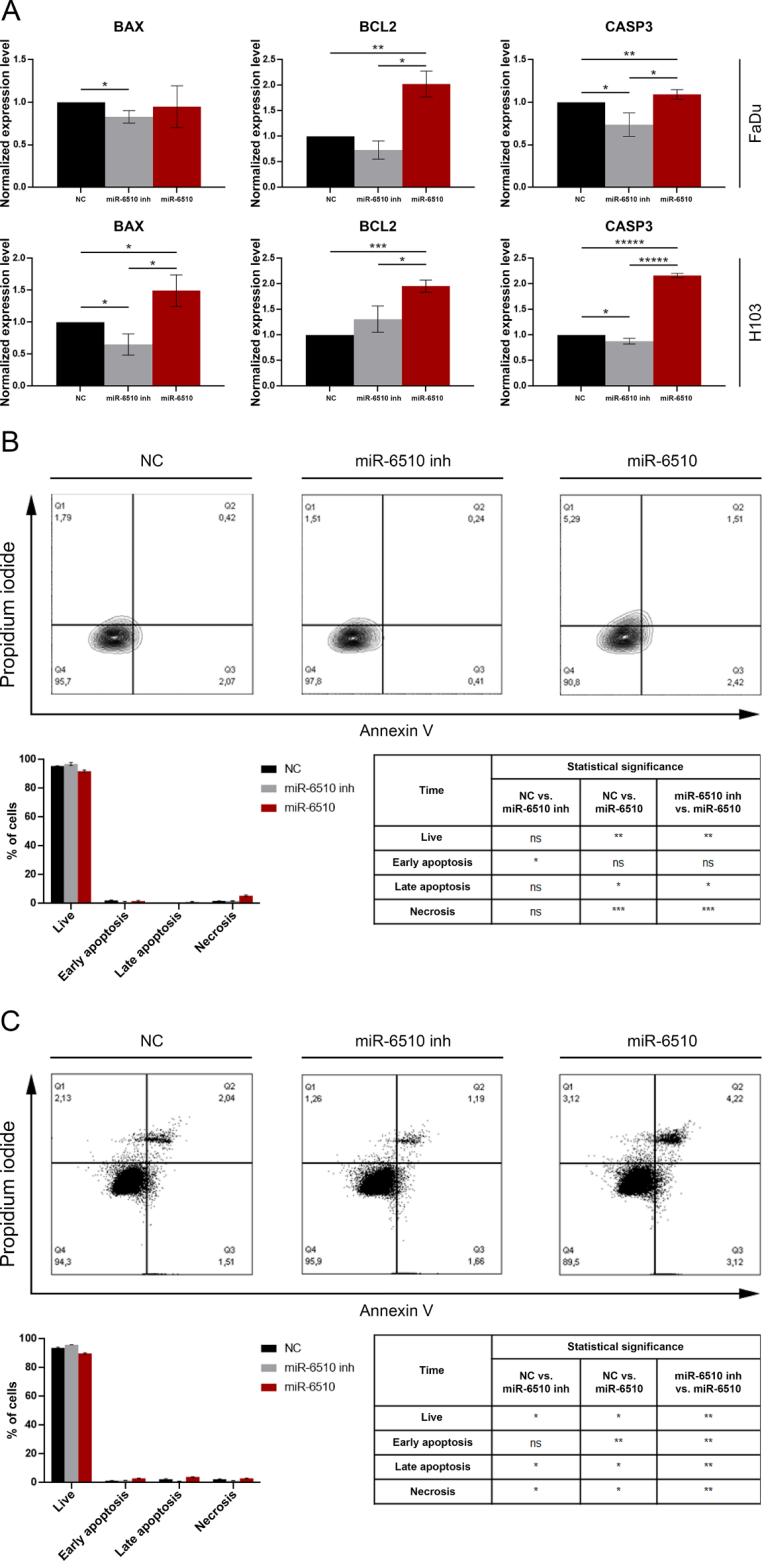
Impact of hsa-miR-6510-3p on apoptotic genes regulation in FaDu and H103 cells. (**A**) Analysis of apoptosis-related genes expression at the transcriptional level with qPCR. Flow cytometry analysis of (**B**) FaDu and (**C**) H103 cell death. Data presented as percentages of cells in the individual cell death stages. NC – cells transfected with non-specific, control miRNA; miR-6510 inh – cells transfected with hsa-miR-6510-3p inhibitor; miR-6510 - cells transfected with hsa-miR-6510-3p. Data are presented as the mean ± standard deviation. *p < 0.05, **p < 0.01, ***p < 0.001, ****p < 0.0001, ****p < 0.00001 with comparisons indicated by lines/in the table.

In order to investigate the effect of hsa-miR-6510-3p on the induction of apoptosis in FaDu and H103 cells, gene expression analysis was performed at the transcript level using RT-qPCR. The analysis was performed on day 5 after miR-6510-3p transfection. The transfection of FaDu cells with hsa-miR-6510-3p caused significant increase od *CASP3* gene when comparing with control and inhibitor transfected cells (*p* = 0.048194 and *p* = 0.036366, respectively). There was also observed in FaDu cells a statistically significant decrease in the expression of the *BAX* gene, the activator of apoptosis, in cells treated with the hsa-miR-6510-3p inhibitor compared to the control cells (*p* = 0.015112) (Fig. 6A).

In the H103 cell line an increase in the expression level of the *CASP3* gene was also observed in cells transfected with hsa-miR-6510-3p in relation to the control variant (*p* = 0.000001) and to the cells treated with the miR-6510-3p inhibitor (*p* = 0.000006). A decrease in the expression of this gene was also observed in cells transfected with the miR-6510-3p inhibitor compared to control cells (*p* = 0.020835). Similar observations were made for the *BAX* gene. An increase in its expression was demonstrated in cells transfected with miR-6510-3p, both compared to the control variant (*p* = 0.025265) and to cells treated with the miR-6510-3p inhibitor (*p* = 0.025590). The reduction in the *BAX* gene expression level was also statistically significant in cells transfected with the miR-6510-3p inhibitor compared to control cells (*p* = 0.029007) (Fig. 6A).

In the case of both cell lines, it was observed that transfection of hsa-miR-6510-3p results in an increase in the expression of the *BCL2* gene, a factor that inhibits the release of cytochrome c and apoptosis-inducing factor (AIF) from the mitochondria at the induction stage of the apoptosis process. It was shown that the expression level of this gene in cells transfected with hsa-miR-6510-3p increases by approximately 100% compared to control cells in both the FaDu (*p* = 0.004481) and H103 cell lines (*p* = 0.0005870). In the case of both tested cell lines, there was also a statistically significant increase in the *BAX* gene expression level in ‘miR-6510-3p’ cells compared to the variant treated with miR-6510-3p inhibitor (FaDu: *p* = 0.027376; H103: *p* = 0.049513) (Fig. 6A).

In order to investigate the effect of hsa-miR-6510-3p on the induction of cell death, the Annexin V/propidium iodide staining and the flow cytometry analysis were also performed on day 7 after cell transfection.

The FaDu cells transfected with miR-6510-3p showed a statistically significant decrease in the percentage of live cells (91.93%) compared to control cells (95.57%; *p* = 0.001754), as well as an increase in the fraction of cells in the late apoptosis (0.45% vs. 1.04%; *p* = 0.039511) and necrosis (1.82% vs. 5.39%; *p* = 0.000314) (Fig. 6B). The study of H103 cells showed that transfection of miR-6510-3p results in a decrease in the fraction of live cells (89.90%) compared to the control variant (93.76%; *p* = 0.015245) and an increase in the percentage of cells in the early apoptosis (1.44% vs. 3.04%; *p* = 0.002500) and late apoptosis (2.41% vs. 4.02%; *p* = 0.031640) stage (Fig. 6C).

### MiR-6510-3p increases cancer cells sensitivity to ionizing radiation and enhances double strand breaks formation

In order to investigate the effect of hsa-miR-6510-3p on the response of head and neck squamous cell carcinoma cells to the ionizing radiation, the cells were irradiated at a dose of 2 Gy and subjected to cytometric analysis of DSBs marker - γH2AX.

**Fig. 7:**
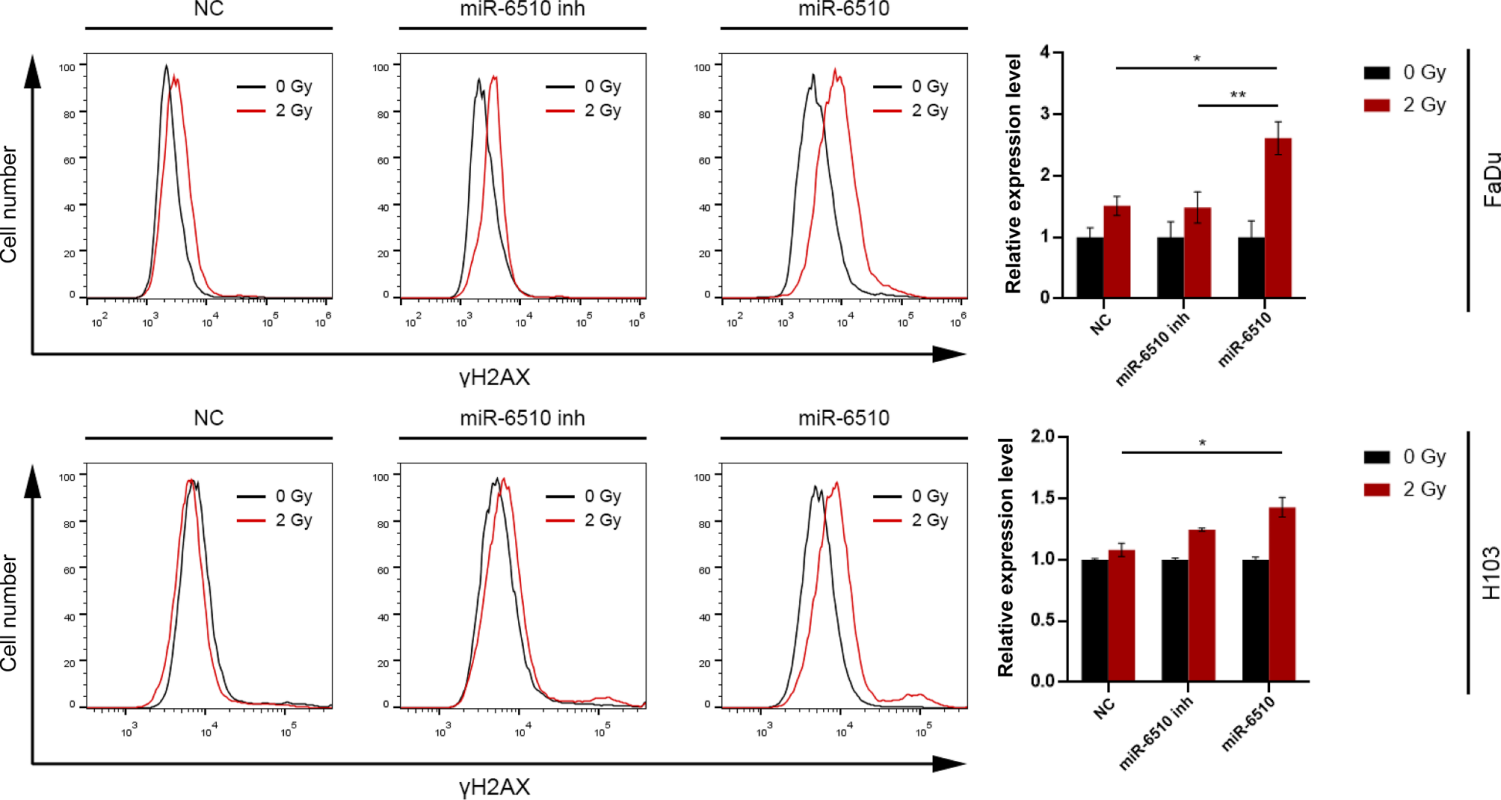
Impact of hsa-miR-6510-3p on FaDu and H103 cells sensitivity to ionizing radiation and Double Strand Breaks formation. Flow cytometry analysis of γH2AX expression. NC – cells transfected with non-specific, control miRNA; miR-6510 inh – cells transfected with hsa-miR-6510-3p inhibitor; miR-6510 - cells transfected with hsa-miR-6510-3p. Data are presented as the mean ± standard deviation. *p < 0.05, **p < 0.01 with comparisons indicated by lines.

In the FaDu cell line a statistically significant increase in the response to radiation was observed in cells treated with hsa-miR-6510-3p both in relation to the control variant (NC, *p* = 0.0258763) and to cells transfected with miR-6510-3p inhibitor (*p* = 0.028343) (Fig. 7). The analysis of H103 cells showed a statistically significant increase in DSBs formation in cells transfected with miR-6510-3p compared to the control variant (*p* = 0.0361516) (Fig. 7).

## Discussion

Presently clinical treatment of cancer includes many layers of diagnosis including detailed molecular analysis of tumor tissue with inclusion of high-throughput next generation sequencing (NGS) allowing in depth understanding the genetics of the malignant changes. Yet, there is an ongoing search for novel and reliable biomarkers for different malignant diseases to improve early detection, prediction of disease outcome and to develop personalized treatment. Blood circulating biomarkers are of particular interest due to easy and minimally invasive procedures allowing frequent collection from patients before during and after the treatment. Our team investigated several different approaches to study miRNAs as potential biomarkers in plasma, tumor, and healthy tissue from the same individual. Over the years our team and other laboratories detected many different miRNAs classified as potential oncomirs, representing miRNAs associated with cancer or the opposite one known as tumor suppressors^16,23–25^. Importantly, our random forest survival analysis of the The Cancer Genome Atlas HNSC dataset from 497 HNSCC patients narrowed the selection of the wide variety of miRNAs to 11 upregulated (i.e., miR-196b-5p, miR-1269a, miR-196a-5p, miR-4652-3p, miR-210-3p, miR-1293, miR-615-3p, miR-503-5p, miR-455-3p, miR-205-5p, and miR-21-5p) and 9 downregulated (miR-376c-3p, miR-378c, miR-29c-3p, miR-101-3p, miR-195-5p, miR-299-5p, miR-139-5p, miR-6510-3p, miR375) which were associated with clinical outcomes^25^. More importantly, optimal random forest analysis including 6 different variables such as age at diagnosis, sex, N stage, pathologic stage, gender, and race brought our attention to one particular miRNA, miR-6510-3p^25^. This was also one of the miRNAs that was not studied in HNSCC and understudied in other malignant types. Additional study by our group using cohort from The Greater Poland Cancer Centre in Poznan, Poland indicated that this miRNA is significantly downregulated in tumor tissue when comparing with histologically normal tissue from the same patient, and, more importantly, the survival analysis indicated that lower expression in tumor tissue predicts lower chances for recovery and survival^24^.

Following these initial data here we tried to determine the role of miR-6510-3p in HNSCC pathogenesis using two established head and neck cancer cell lines: H103 and FaDu. The PCR analysis of miR-6510-3p expression indicated that single transfection of both cell lines with the mimic of this miRNA caused significant elevation when measured after 5 and 7 days. This data shows that transfecting HNSCC cell lines with specific miRNA mimic allows long-term upregulation of studied molecule allowing further functional studies to determine the role of this miRNA of tumor’s cell function.

Importantly, follow-up analysis using flow cytometry indicated that increasing the level of miR-6510-3p through the transfection with the mimic significantly induced the process of apoptosis of these tumor cells and shifted the populations of the cells by increasing the percentage of cells in G2/M phase vs. lower percentage of G1 phase. This was supported by qPCR gene expression analysis, which revealed increased levels of the pro-apoptotic genes *BAX* and *CASP3* in cells transfected with miR-6510-3p. Interestingly, these cells also exhibited increased expression of the anti-apoptotic gene *BCL-2*. According to Michaud et al., in HNSCC, increased *BCL-2* expression often correlates with chemoresistance and tumor survival, while *BAX* activation may represent a cellular attempt to induce apoptosis in response to therapeutic stress. However, the overexpression of *BCL-2* can antagonize *BAX*, allowing cancer cells to evade apoptosis and continue proliferating^26^. It was also indicated that the tumor suppressor protein p53 activates the expression of the *BAX* gene, leading to the induction of apoptosis. However, *BAX* can also influence p53 function, creating a feedback loop in which both proteins regulate each other’s expression, further complicating the mechanisms controlling apoptosis^27^. As demonstrated in several studies, the intricate interplay and balance between *BAX* and *BCL-2* is critical in regulating apoptosis and plays a pivotal role in the pathophysiology of head and neck cancer.

Observed increase in apoptosis with concomitant shifts in population of G2/M phase suggest that increased level of miR-6510-3p in both FaDu and H103 cells activate the mechanism promoting cellular death or cell cycle arrest, one of desire mechanism protecting from tumor growth and development^28^. Promoting cell cycle arrest or apoptosis might indicate one of the mechanisms by which individuals with higher expression level of miR-6510-3p in tumor tissue have greater chances for recovery and survival as observed in clinical studies^24,25^. Also MTT assay for proliferation and wound healing test for migration showed that targeting HNSCC cell lines with miR-6510-3p is critical in reducing the tumor growth and potential tumor cell migration decreasing the risk of metastasis as observed in low expressing cells.

Beside shifting the cell cycle populations, activating apoptosis which was reflected in lower proliferation rate and suppressed migration our analysis also indicated that miR-6510-3p reduced CD133 marker in both H103 and FaDu cells suppressing stemness of these cells. Overall, different studies in varied type of tumors correlate CD133 with resistance to chemotherapy, while low CD133 expression predict better responses^29^. It was also supported by the Zhang et al., who showed that isolated cancer stem cells (CSCs) from human oral squamous cell carcinoma that are CD133 positive showed substantially higher resistance to standard chemotherapy^30^. Our study demonstrated that miR-6510-3p reduces colony formation ability in head and neck cancer cells. Han et al. indicated that high clonogenic potential of CSCs in HNSCC plays a crucial role in tumor initiation, progression, recurrence, and therapy resistance^31^. It was also supported by Schniewind, et al.^32^and Yaromina et al., who indicated that high clonogenic potential is associated with increased tumor aggressiveness and resistance to conventional therapies, such as chemotherapy and radiation^33^. Yet, our analysis indicated that increasing miR-6510-3p is also critical in increasing the sensitivity to HNSCC cells to radiation, the major treatment provided to HNSCC patients.

This initial in vitro study supports the importance of miR-6510-3p in HNSCC pathogenesis. Overall, the functional studies showed that despite significant value as a biomarker this strongly suppressed miRNA in HNSCC tumor tissue might represent one of the key therapeutic components that should be considered as an addition to future personalized/direct therapy. Importantly, despite limited techniques allowing direct targeting of specific tissues i.e., HNSCC tumor with miRNA, there are already ongoing clinical trials against human disease, including hepatitis C and certain cancers^34^. However, with moving the development of new target delivery there is hope in close future that the medicine will be able to target directly different types of cells before, during and after therapy. Yet, functional studies like this will be critical to better understand the function of specific selected miRNAs to design novel clinical trials based on miRNA targeted therapies and improve patients’ outcomes.

The present study has some limitations. To gain a better understanding of the mechanisms of action of miR-6510-3p and to identify the signaling pathways regulated by this molecule, it is essential to conduct extensive analyses using high-throughput methods. Furthermore, to confirm the role of miR-6510-3p in the pathogenesis of HNSCC, future in vivo experiments are necessary to validate observed changes in animal model. In the present study, the control variant consists of HNSCC cells transfected with non-specific, control miRNA. Vehicle control (cells treated only with the transfection reagent) was not used in this work, which may also represent a potential limitation of the study.

In conclusion, our study indicated the importance of miR-6510-3p not only as a valuable marker of the disease but also as a potential driver of HNSCC. We observed that transfection of HNSCC cells with hsa-miR-6510-3p causes the cell cycle arrest in G2/M phase and is associated with a decrease of cell proliferation and migration ability of cancer cells. We have also demonstrated that hsa-miR-6510-3p induces cell death, increases the sensitivity of HNSCC cells to ionizing radiation and causes a loss of the stemness properties responsible for the occurrence of metastases and relapses of the disease. These results indicated the importance of miR-6510-3p as a marker and a driver of HNSCC disease and increasing its levels or potentially only normalizing it can increase the chances for much more effective treatment and full recovery from the disease.

## Methods

### Cell culture

In order to investigate the influence of the microRNA hsa-miR-6510-3p on HNSCC cell biology, two different TP53-mutant cancer cell lines were cultured: FaDu (ATCC^®^ HTB-43™) cell line established from hypopharyngeal tumor (kindly provided by Prof. Michael Baumann, OncoRay - National Center for Radiation Research in Oncology, Technische Universität Dresden) and H103 cell line established from a small squamous cell carcinoma (SSC) of the tongue, obtained from European Collection of Authenticated Cell Cultures (ECACC, 06092001).

Both cell lines were at passage 9 when used in the study. Cells were maintained at 37 °C, 5% CO_2_ atmosphere and a humidity level of 95%. FaDu cell line was cultured in DMEM (Biowest, France) medium supplemented with 10% fetal bovine serum (FBS) (Biowest, France) and 1% penicillin/streptomycin (Biowest, France). H103 cells were cultured in DMEM/Ham’s F12 (1:1) medium (Biowest, France) supplemented with 10% FBS, 1% penicillin/streptomycin and 1% L-glutamine (Biowest, France).

### Transfection

MiR-6510-3p mimic (cat. no. 4464066), miR-6510-3p inhibitor (cat. no. 4464084) and miRNA negative control (cat. no. 4464059) were purchased from Thermo Fisher Scientific (MA, USA). Transfection was performed using Lipofectamine^®^ RNAiMAX Transfection Reagent (Thermo Scientific, MA, USA) and Opti-MEM^®^ serum-free medium (Thermo Scientific, MA, USA) according to the protocol provided by the manufacturer. Briefly, 3 × 10^5^ cells were seeded into 6-well plates one day prior to transfection. When the cells reached 50–70% confluency, miRs were transfected into the cells as miRNA-lipid complexes at a final concentration of 20 nM.

### RT-qPCR analysis of mir-6510-3p expression level

Total RNA (including miRNAs fraction) was extracted at 5th and 7th day after transfection with hsa-miR-65103-3p mimic, inhibitor and negative control using QIAzol Lysis Reagent (Qiagen, Germany) and miRNeasy Mini Kit (Qiagen, Germany) according to manufacturer’s protocol. RNA concentration and quality were measured (absorbance at 260, 230, and 280 nm) using DeNovix DS-11 spectrophotometer (DeNovix, Wilmington, DE, USA). cDNA template was prepared using TaqMan™ Advanced miRNA cDNA Synthesis Kit (Applied Biosystems, Waltham, MA, USA) according to manufacturer’s instructions with 10 ng of total RNA input. For the RT-qPCR reaction a 10-fold dilution of cDNA template was prepared. RT-qPCR was performed using the LightCycler^®^96 system (Roche, Germany), TaqMan™ Fast Advanced Master Mix (Applied Biosystems, Waltham, MA, USA) and TaqMan™ Advanced miRNA Assays with primers specific for selected target miRNAs: hsa-miR-6510-3p (Assay ID: 480744_mir), hsa-miR-16-5p (Assay ID: 477860_mir) (Table 1). Each reaction was performed in three technical replicates. The 2−∆∆Ct method was used to calculate the miRNA expression level. The hsa-miR-16-5p microRNA served as an endogenous internal control and was used for RT-qPCR results normalization.

**Table 1:** Stem-loop primers used for miRNA expression analysis.

miRNA	Mature miRNA sequence	Stem-loop primer sequence	miRBase Accession Number
hsa-miR-6510-3p	CACCGACUCUGUCUCCUGCAG	AGCAGCAGGGGAGAGAGAGGAGUCCUCUAGACACCGACUCUGUCUCCUGCAGAU	MIMAT0025477
hsa-miR-16-5p	UAGCAGCACGUAAAUAUUGGCG	GUCAGCAGUGCCUUAGCAGCACGUAAAUAUUGGCGUUAAGAUUCUAAAAUUAUCUCCAGUAUUAACUGUGCUGCUGAAGUAAGGUUGAC	MIMAT0000069

### Cell cycle analysis with flow cytometry

Cell cycle analysis was performed at 5th and 7th day after transfection with hsa-miR-65103-3p mimic, inhibitor and negative control as previously described^35^. Briefly, after treatment with miR-6510-3p mimic, miR-6510-3p inhibitor and negative control miRNA, cells were harvested by trypsinization, washed twice with 1 mL PBS and fixed with 70% ethanol overnight at − 20 °C. After the incubation cells were washed twice with PBS and centrifuged (5 min, 3000 rpm). Cell pellet was suspended in 200 µl of solution containing 50 µg/mL propidium iodide (Cayman Chemicals, MI, USA) and 10 µg/mL RNase A (AppliChem, Germany) in PBS, incubated for 30 min at 37 °C in the dark and then analyzed with a CytoFLEX flow cytometer (Beckman Coulter, CA, USA). The percentages of cells in the G1, S, and G2-M phases were determined using FlowJo v10.6.1 software (FlowJo, LLC, OR, USA; https://www.flowjo.com/).

### Cell proliferation analysis

Cell proliferation was evaluated at 7th day after transfection with hsa-miR-65103-3p mimic, inhibitor and negative control using MTT assay. Briefly, HNSCC cells were plated in a 96-well plate at a concentration of 2.0 × 10^3^ cells/well for FaDu cell line and 1.5 × 10^3^ cells/well for H103 cells and cultured for 72 h. Then, the culture medium was replaced with one containing 0.5 mg/ml of MTT (Thermo Scientific, MA, USA). Cells were incubated for 2 h under standard conditions. After the incubation, the reagent was replaced with 100 µl of DMSO (dimethyl sulfoxide) (Sigma-Aldrich, MA, USA) in order to suspend developed formazan crystals. The plate was incubated at 37 °C for 10 min and absorbance was measured at 570 nm using Multiskan™ FC Microplate Photometer (Thermo Scientific, MA, USA). Results were normalized to absorbance level of the cells transfected with non-specific negative control miRNA. The relative cell proliferation was calculated using the following formula:

$$\:Proliferation\:\left[\%\right]=\frac{{A}_{x}-{A}_{0}}{{A}_{ctr}-{A}_{0}}\times\:100\%,$$

where: A_x_ is the absorbance the test sample, A0 is the absorbance of the blank sample (non-treated with MTT), A_ctr_ is the absorbance of the control sample.

### Cell migration assay in vitro

Head and neck cancer cells motility was analyzed at 5th day after transfection with the wound healing assay. Briefly, FaDu and H103 cells were seeded in a 12-well culture plate, transfected and cultured for 4 days to form a confluent monolayer. In order to reduce cell proliferation, for 20 h prior to wounding the cells were maintained in serum-free culture medium. A 200 µl pipette tip was used to create the scratch. Cell migration was observed and documented at 12-hour (H103 cell line) and 24-hour (FaDu cell line) intervals using the Zeiss Axio Vert.A1 microscope (Zeiss, Germany). The results were analyzed with ImageJ (version 1.54i) software (National Institutes of Health, NY, USA; https://imagej.net/ij/). The percentage of wound healing was calculated using the following formula:

$$\:C=\frac{{A}_{0}-{A}_{t}}{{A}_{0}}\times\:100\%,$$

where: A_0_ is the initial wound area, A_t_ is the wound area at indicated time t.

### Flow cytometry analysis of stem cell phenotype markers

HNSCC cells were harvested at 7th day after transfection with hsa-miR-65103-3p mimic, inhibitor and negative control and washed twice with PBS. Cell pellet was suspended with 90 µl of 1% BSA solution in PBS. 5 µl of APC-conjugated mouse anti-CD44 antibody (cat. #559942, BD Biosciences, CA, USA) and 5 µl of PE-conjugated mouse anti-CD133 antibody (cat. #566593, BD Biosciences, CA, USA) was added and incubated for 30 min at 4 °C in the dark. Cells were washed twice in PBS and acquired using CytoFLEX flow cytometer (Beckman Coulter, CA, USA). The results were analyzed with FlowJo v10.6.1 software (FlowJo, LLC, OR, USA; https://www.flowjo.com/).

### Clonogenic assay

Colony-forming ability of HNSCC cells was determined with clonogenic assay. Briefly, at 5th day after transfection with hsa-miR-65103-3p mimic, inhibitor and negative control cells were seeded in a 6-well plate at a concentration of 1.2 × 10^3^ cells/well for FaDu cell line and 0.6 × 10^3^ cells/well for H103 cell line, and cultured for 7 days. At day 7 each well was washed with PBS and fixed with absolute methanol at room temperature for 15 min. Cells were stained with 0.1% solution of Coomassie Brilliant Blue R-250 (Bio-Rad, CA, USA) in methanol: acetic acid: water, 5:10:4 (v/v/v) at room temperature for 15 min, washed twice with water and dried at room temperature overnight. Plates were scanned with ChemiDoc™ Touch Gel Imaging System (Bio-Rad, CA, USA). Colonies were counted using ImageJ (version 1.54i) software (National Institutes of Health, NY, USA; https://imagej.net/ij/).

### RT-qPCR analysis of apoptotic genes expression

The analysis of gene expression was performed at 5th day after transfection using qPCR. Briefly, cDNA was synthesized with iScript cDNA Synthesis Kit (Bio-Rad, CA, USA) using 1000 ng of total RNA, oligo dT primers, and random hexamer primers. For the qPCR reaction a 10-fold dilution of cDNA template was prepared. qPCR was performed using the LightCycler^®^96 system (Roche, Germany) and specific primers (Table 2) designed with Universal Probe Library software (Roche Diagnostics, Switzerland). Amplification products were detected with fluorescent probes (Universal Probe Library, Roche, Germany) (Table 2). Each reaction was performed in three technical replicates. The 2−∆∆Ct method was used to calculate the gene expression level. The expression was normalized to the Glyceraldehyde 3-phosphate dehydrogenase (*GAPDH*) housekeeping gene.

**Table 2:** Primers and probes used for gene expression analysis.

Gene	Forward primer	Reverse primer	Probe
*CASP3*	TTGTGGAATTGATGCGTGAT	GGCTCAGAAGCACACAAACA	#68
*BAX*	ATGTTTTCTGACGGCAACTTC	ATCAGTTCCGGCACCTTG	#57
*BLC2*	GCACCTGCACACCTGGAT	AGCCAGGAGAAATCAAACAGAG	#57
*GAPDH*	TCCACTGGCGTCTTCACC	GGCAGAGATGATGACCCTTTT	#45

### Cell death analysis by flow cytometry

The analysis was performed at 5th and 7th day after transfection with FITC Annexin V Apoptosis Detection Kit I (BD Biosciences, NJ, USA) according to the manufacturer protocol. Briefly, 10^5^ cells were washed twice with PBS and treated with 5 µl of APC-conjugated Annexin V and 5 µl of propidium iodide. Cells were incubated for 15 min at room temperature in the dark and then acquired with CytoFLEX flow cytometer (Beckman Coulter, CA, USA). The results were analyzed using FlowJo v10.6.1 software (FlowJo, LLC, OR, USA; https://www.flowjo.com/).

### Flow cytometry analysis of DNA double-strand breaks

HNSCC cells were irradiated with 2 Gy dose using Gammacell^®^ 1000 Elite (BestTheratronics Ltd, Canada) apparatus, harvested and stained with anti-γH2AX antibody according to manufacturer protocol (Apoptosis, DNA Damage and Cell Proliferation Kit; Becton Dickinson, NJ, USA). Briefly, cells were fixed with BD Cytofix/Cytoperm Fixation/Permeabilization Solution (30 min, room temperature), then permeabilized with BD Cytofix/Cytoperm Plus Permeabilization Buffer for 10 min at 4 °C, and re-fixed with BD Cytofix/Cytoperm Fixation/Permeabilization Solution (5 min, room temperature). Subsequently, cells were washed and stained with APC-conjugated mouse anti-γH2AX antibody for 20 min in the dark. After incubation cells were washed with BD Perm/Wash Buffer and analyzed using CytoFLEX flow cytometer (Beckman Coulter, CA, USA). The γH2AX level was determined with FlowJo v10.6.1 software (FlowJo, LLC, OR, USA; https://www.flowjo.com/).

### Statistical analysis of the data

All experiments were performed in triplicate. Two-tailed unpaired Student’s t test, Mann-Whitney test, and the one-way analysis of variance (ANOVA) were performed using GraphPad Prism (version 8.0) software (GraphPad Software, CA, USA; https://www.graphpad.com/). For multiple comparisons post-hoc Tukey’s test was performed. P values of less than 0.05 were considered statistically significant and are indicated by the (*) symbol for *p* < 0.05, by (**) for *p* < 0.01, by (***) for *p* < 0.001, by (****) for *p* < 0.0001, or by (*****) for *p* ≤ 0.00001.

### Data Availability

The datasets used and/or analyzed during the current study are available from the corresponding author on reasonable request.

## References

[1] Bray, F. et al. Global cancer statistics 2018: GLOBOCAN estimates of incidence and mortality worldwide for 36 cancers in 185 countries. *CA Cancer J. Clin.* **68**, 394–424 (2018). 30207593 10.3322/caac.21492

[2] Sanderson, R. J. Squamous cell carcinomas of the head and neck * Commentary: Head and neck carcinomas in the developing world. *BMJ* **325**, 822–827 (2002). 12376446 10.1136/bmj.325.7368.822 PMC1124330

[3] Thomas, S. M. & Grandis, J. R. The current state of Head and Neck Cancer Gene Therapy. *Hum. Gene Ther.* **20**, 1565–1575 (2009). 19747066 10.1089/hum.2009.163 PMC2829451

[4] Maiti, G. P. et al. Overexpression of EGFR in Head and Neck squamous cell carcinoma is Associated with inactivation of SH3GL2 and CDC25A genes. *PLoS One*. **8**, e63440 (2013). 23675485 10.1371/journal.pone.0063440 PMC3651136

[5] Martinez-Useros, J. & Garcia-Foncillas, J. The challenge of blocking a wider family members of EGFR against head and neck squamous cell carcinomas. *Oral Oncol.* **51**, 423–430 (2015). 25753560 10.1016/j.oraloncology.2015.02.092

[6] Johnson, D. E. et al. Head and neck squamous cell carcinoma. *Nat. Rev. Dis. Primers*. **6**, 92 (2020). 33243986 10.1038/s41572-020-00224-3 PMC7944998

[7] Leemans, C. R., Snijders, P. J. F. & Brakenhoff, R. H. The molecular landscape of head and neck cancer. *Nat. Rev. Cancer*. **18**, 269–282 (2018). 29497144 10.1038/nrc.2018.11

[8] Friedman, R. C., Farh, K. K. H., Burge, C. B. & Bartel, D. P. Most mammalian mRNAs are conserved targets of microRNAs. *Genome Res.* **19**, 92–105 (2009). 18955434 10.1101/gr.082701.108 PMC2612969

[9] Li, Y. & Kowdley, K. V. MicroRNAs in Common Human diseases. *Genomics Proteom. Bioinf.* **10**, 246–253 (2012). 10.1016/j.gpb.2012.07.005 PMC3611977 23200134

[10] Li, N., Long, B., Han, W., Yuan, S. & Wang, K. microRNAs: important regulators of stem cells. *Stem Cell. Res. Ther.* **8**, 110 (2017). 28494789 10.1186/s13287-017-0551-0 PMC5426004

[11] Victoria, B., Lopez, N., Masternak, M. M. & Y. O. & MicroRNAs and the metabolic hallmarks of aging. *Mol. Cell. Endocrinol.* **455**, 131–147 (2017). 28062199 10.1016/j.mce.2016.12.021 PMC5724961

[12] Puram, S. V. & Rocco, J. W. Molecular aspects of Head and Neck Cancer Therapy. *Hematol. Oncol. Clin. North. Am.* **29**, 971–992 (2015). 26568543 10.1016/j.hoc.2015.07.003 PMC4648693

[13] Huang, Y. et al. Construction of an 11-microRNA-based signature and a prognostic nomogram to predict the overall survival of head and neck squamous cell carcinoma patients. *BMC Genom.* **21**, 691 (2020). 10.1186/s12864-020-07104-w PMC7542341 33023466

[14] Ayaz, L., Görür, A., Yaroğlu, H. Y., Özcan, C. & Tamer, L. Differential expression of microRNAs in plasma of patients with laryngeal squamous cell carcinoma: potential early-detection markers for laryngeal squamous cell carcinoma. *J. Cancer Res. Clin. Oncol.* **139**, 1499–1506 (2013). 23817697 10.1007/s00432-013-1469-2 PMC11824634

[15] Kikkawa, N. et al. miR-489 is a tumour-suppressive miRNA target PTPN11 in hypopharyngeal squamous cell carcinoma (HSCC). *Br. J. Cancer*. **103**, 877–884 (2010). 20700123 10.1038/sj.bjc.6605811 PMC2966617

[16] Martinez, B. V. et al. Circulating small non coding RNA signature in head and neck squamous cell carcinoma. *Oncotarget* **6**, 19246–19263 (2015). 26057471 10.18632/oncotarget.4266 PMC4662488

[17] Scapoli, L. et al. MicroRNA expression profiling of oral carcinoma identifies new markers of Tumor Progression. *Int. J. Immunopathol. Pharmacol.* **23**, 1229–1234 (2010). 21244772 10.1177/039463201002300427

[18] Gombos, K. et al. miRNA expression profiles of oral squamous cell carcinomas. *Anticancer Res.* **33**, 1511–1517 (2013). 23564792

[19] Chen, X., Liang, H., Zhang, J., Zen, K. & Zhang, C. Y. Secreted microRNAs: a new form of intercellular communication. *Trends Cell. Biol.* **22**, 125–132 (2012). 22260888 10.1016/j.tcb.2011.12.001

[20] Corrado, C. et al. Exosomes as Intercellular Signaling Organelles Involved in Health and Disease: Basic Science and clinical applications. *Int. J. Mol. Sci.* **14**, 5338–5366 (2013). 23466882 10.3390/ijms14035338 PMC3634447

[21] Schneider, A. & Simons, M. Exosomes: vesicular carriers for intercellular communication in neurodegenerative disorders. *Cell. Tissue Res.* **352**, 33–47 (2013). 22610588 10.1007/s00441-012-1428-2 PMC3602607

[22] Ahmed, K. A. & Xiang, J. Mechanisms of cellular communication through intercellular protein transfer. *J. Cell. Mol. Med.* **15**, 1458–1473 (2011). 20070437 10.1111/j.1582-4934.2010.01008.x PMC3823191

[23] Schneider, A. et al. Tissue and serum microRNA profile of oral squamous cell carcinoma patients. *Sci. Rep.* **8**, 675 (2018). 29330429 10.1038/s41598-017-18945-z PMC5766573

[24] Piotrowski, I. et al. miRNAs as biomarkers for Diagnosing and Predicting Survival of Head and Neck squamous cell carcinoma patients. *Cancers (Basel)*. **13**, 3980 (2021). 34439138 10.3390/cancers13163980 PMC8392400

[25] Nunez Lopez, Y. O., Victoria, B., Golusinski, P., Golusinski, W. & Masternak, M. M. Characteristic miRNA expression signature and random forest survival analysis identify potential cancer-driving miRNAs in a broad range of head and neck squamous cell carcinoma subtypes. *Rep. Practical Oncol. Radiotherapy*. **23**, 6–20 (2018). 10.1016/j.rpor.2017.10.003 PMC5698002 29187807

[26] Michaud, W. A. et al. Bcl-2 blocks cisplatin-induced apoptosis and predicts poor outcome following chemoradiation in advanced oropharyngeal squamous cell carcinoma. *Clin. Cancer Res.* **15**, 1645–1654 (2009). 19240170 10.1158/1078-0432.CCR-08-2581 PMC2745309

[27] Wei, H. et al. Structures of p53/BCL-2 complex suggest a mechanism for p53 to antagonize BCL-2 activity. *Nat. Commun.* **14**, 4300 (2023). 37463921 10.1038/s41467-023-40087-2 PMC10353994

[28] Stark, G. R. & Taylor, W. R. Control of the G2/M transition. *Mol. Biotechnol.* **32**, 227–248 (2006). 16632889 10.1385/MB:32:3:227

[29] Li, Z. CD133: a stem cell biomarker and beyond. *Exp. Hematol. Oncol.* **2**, 17 (2013). 23815814 10.1186/2162-3619-2-17 PMC3701589

[30] Zhang, Q. et al. A subpopulation of CD133 + cancer stem-like cells characterized in human oral squamous cell carcinoma confer resistance to chemotherapy. *Cancer Lett.* **289**, 151–160 (2010). 19748175 10.1016/j.canlet.2009.08.010

[31] Han, J. et al. Identification and characterization of cancer stem cells in human head and neck squamous cell carcinoma. *BMC Cancer*. **14**, 173 (2014). 24612587 10.1186/1471-2407-14-173 PMC4008349

[32] Schniewind, I. et al. Epigenetic targeting to overcome radioresistance in head and neck cancer. *Cancers* **16**, 730 (2024). 38398123 10.3390/cancers16040730 PMC10886471

[33] Yaromina, A. et al. Pre-treatment number of clonogenic cells and their radiosensitivity are major determinants of local tumour control after fractionated irradiation. *Radiother Oncol.* **83**, 304–310 (2007). 17517444 10.1016/j.radonc.2007.04.020

[34] Chakraborty, C., Sharma, A. R., Sharma, G., Doss, C. G. P. & Lee, S. S. Therapeutic miRNA and siRNA: moving from Bench to Clinic as Next Generation Medicine. *Mol. Ther. Nucleic Acids*. **8**, 132–143 (2017). 28918016 10.1016/j.omtn.2017.06.005 PMC5496203

[35] Barczak, W. et al. hTERT gene knockdown enhances response to radio- and chemotherapy in head and neck cancer cell lines through a DNA damage pathway modification. *Sci. Rep.* **8**, 5949 (2018). 29654294 10.1038/s41598-018-24503-y PMC5899166

